# Anxiety and Depression Among Hepatitis B Inpatients in Shenzhen, China: A Cross-Sectional Study

**DOI:** 10.31083/AP49373

**Published:** 2025-12-23

**Authors:** Yanping Chen, Gang Duan, Tao Wang, Xinying Wang, Ting He, Tiantian Liu, Ying He, Xiaoning Liu, Hongzhou Lu

**Affiliations:** ^1^Center for Hepatology Medicine, Shenzhen Third People’s Hospital and The Second Affiliated Hospital of Southern University of Science and Technology, 518112 Shenzhen, Guangdong, China; ^2^Department of Infection and Immunology, Shenzhen Third People’s Hospital and The Second Affiliated Hospital of Southern University of Science and Technology, 518112 Shenzhen, Guangdong, China; ^3^National Clinical Research Center for Infectious Disease, 518112 Shenzhen, Guangdong, China

**Keywords:** chronic hepatitis B, anxiety, depression, inpatients, Hospital Anxiety and Depression Scale (HADS)

## Abstract

**Background:**

Chronic hepatitis B (CHB) represents a significant global public health challenge. In China, the disease remains prevalent despite recent reductions in incidence. In addition to its impact on physical health, CHB adversely affects patients’ mental health, particularly in the form of anxiety and depression. However, limited research has been conducted on the psychological status of CHB inpatients, especially in metropolitan settings. This study aimed to evaluate the prevalence of anxiety and depression among hospitalized CHB patients in Shenzhen, China, and to investigate factors associated with these mental health conditions.

**Methods:**

A cross-sectional study was conducted involving 649 inpatients with chronic hepatitis B at Shenzhen Third People’s Hospital. The Hospital Anxiety and Depression Scale (HADS) was used to assess levels of anxiety and depression. Logistic regression analysis was performed to identify factors associated with mental health outcomes.

**Results:**

The study revealed that 34.05% of patients experienced anxiety, while 71.65% exhibited symptoms of depression. Depression was more prevalent among older patients and those with multiple hospitalizations. Factors such as lack of health insurance and prolonged hospitalizations were significantly associated with depression. Female patients showed a higher propensity for experiencing anxiety.

**Conclusion:**

The high prevalence of anxiety and depression among CHB inpatients highlights the need for integrated mental health screening and intervention strategies within hospital settings. Tailored healthcare approaches are essential to address both the physical and psychological needs of CHB patients, particularly in rapidly urbanizing areas such as Shenzhen.

## Main Points

1. High Prevalence of Mental Health Disorders: Among hospitalized Chronic hepatitis B (CHB) patients, 34.1% exhibited anxiety and 71.7% exhibited depressive symptoms, highlighting the significant mental health burden in this cohort.

2. Key Risk Factors for Depression and Anxiety: Depression correlates with advanced age, recurrent hospitalizations, absence of health insurance, and protracted hospital admissions. Women exhibit a greater tendency to worry than men.

3. Minimal Impact of Clinical Parameters: Clinical markers, including liver function tests and dietary indicators, exhibited no significant correlation with anxiety or depression, highlighting the primacy of psychosocial factors.

4. Urban-Specific Stressors: The psychological load is intensified for uninsured patients in Shenzhen due to urban living, financial demands, and stigma associated with CHB.

5. Need for Comprehensive Care: The findings underscore the necessity of incorporating mental health screening and interventions into the treatment of CHB inpatients to effectively address both physical and psychological requirements.

## 1. Introduction

Chronic hepatitis B (CHB) infection constitutes a significant global public health issue, greatly influencing morbidity and mortality rates globally. In 2022, the World Health Organization (WHO) recognized viral hepatitis as a predominant cause of mortality among infectious diseases, exceeded only by COVID-19 and tuberculosis [1]. In 2022, it is estimated that approximately 254 million individuals were afflicted with chronic hepatitis B, with 1.2 million new infections occurring annually [2]. In China, which represents a substantial portion of the global CHB population, the prevalence of hepatitis B remains very high, although there has been a declining trend over the past three decades. Recent meta-analyses reveal that the prevalence of Hepatitis B Virus (HBV) infection in China decreased to roughly 3% between 2018 and 2020, categorizing the country as a lower intermediate epidemic region [3, 4]. Nevertheless, prevalence rates remain elevated in rural areas and western regions, indicating persistent geographical disparities [5, 6, 7].

CHB’s consequences extend beyond physical health, significantly impacting individuals’ mental well-being. Mental health disorders, such as anxiety and depression, frequently occur in individuals diagnosed with CHB, often intensified by social stigmatization, discrimination, and the chronic nature of the condition. These psychosocial disorders are intensified by factors such as alcohol drinking and inadequate social support, which have been shown to negatively impact mental health outcomes in this population [8, 9]. A cross-sectional study by Liu *et al*. [10] revealed a significant association between self-awareness of hepatitis B and depression in the Chinese community, highlighting the substantial mental health burden associated with the condition.

Unlike the hepatitis C virus (HCV), which can be cured with interferon therapy, CHB is often still a chronic disease with serious liver complications such as cirrhosis and hepatocellular carcinoma [11], and the challenge of psychological well-being poses a challenge to the patient. Despite the decreasing prevalence of HBV in China, the social consequences of the disease, including stigmatization and economic burden, continue to exacerbate the mental health issues encountered by patients [12, 13].

Psychological theories, notably the Transactional Model of Stress and Coping [14], provide a beneficial structure for understanding the psychological impacts of chronic disorders like CHB. This paradigm suggests that individuals with chronic illnesses, such as HBV, may view their situations as threatening, leading to emotional responses such as fear and grief. Coping strategies, such as seeking social support or utilizing avoidance behaviors, may influence the severity of these mental health impacts. The Biopsychosocial Model [15] posits that health outcomes are influenced by biological, psychological, and social determinants. This concept advocates for holistic care that tackles the mental health issues faced by CHB patients with their physical health needs.

Additionally, while the mental burden and its effects on health-related quality of life (HRQOL) have been extensively studied in patients with chronic hepatitis C (CHC), there is a surprising paucity of research focused on similar issues in patients with CHB. Furthermore, current research on CHB frequently involve relatively small sample sizes and is performed in outpatient environments, thereby neglecting the inpatient context. Inpatient status frequently signifies a more severe disease condition and more economic burden, while the unfamiliar hospital setting, along with discomfort from treatment measures like venipuncture and liver puncture, may exacerbate mental health issues.

Furthermore, while the prevalence of HBV displays geographic disparities within China, the mental health status of affected individuals across different locations remains largely unexamined. Shenzhen, a highly developed city in China, presents a distinctive setting where socioeconomic considerations, healthcare infrastructure, and patient demography can profoundly impact the mental health outcomes of HBV patients, especially among inpatients.

A notable deficiency exists in research addressing the mental health burden faced by inpatients, especially in urban environments such as Shenzhen. This study intends to examine the anxiety and depression levels among inpatients with hepatitis B, exploring the psychosocial factors that contribute to these mental health disorders and their implications for patient management. This study posits that inpatients with HBV endure a heightened mental health burden. By examining the nexus of chronic hepatitis B and mental health in an inpatient environment, it aims to fill the research void and underscore the necessity for integrated care strategies that cater to both the physical and mental health requirements of CHB inpatients in urban contexts.

## 2. Methods

### 2.1 Study Setting and Participants

This study enlisted volunteers from the Shenzhen Third People’s Hospital, situated in the heart of Shenzhen, southern China. The hospital, a comprehensive facility, has been one of the early HBV-designated hospitals in Shenzhen since 1985, offering high-level HBV care to over 20,000 patients. Annually, approximately 3000 patients receive treatment in the HBV department for HBV-related comorbidities and chronic hepatitis B therapy. Consequently, the participants in this study accurately reflect the population of CHB in Shenzhen, China.

Recruitment for this study was conducted over 15 months, from May 2023 to July 2024, by using non-probability sampling. All participants who met the following inclusion criteria were eligible for participation: (1) HBsAg test was positive; (2) hospitalization in the HBV department in the Third People’s Hospital of Shenzhen; (3) 18 years old and older; and (4) provision of written informed consent. To ensure the robustness and representativeness of the sample, the following exclusion criteria were applied to participants in this study: (1) severe cognitive impairment; (2) severe psychiatric disorders; (3) pregnancy or breastfeeding (to eliminate potential confounding effects of hormonal changes on mental health); (4) history of alcohol or substance abuse; (5) incapacity to communicate or comprehend Chinese. This study enlisted 765 participants, ultimately including 649 in the analysis due to missing data from other individuals.

### 2.2 Measures

Eligible participants were asked to provide written informed consent. Research nurses collected patient data and administered the survey. Participants received CHB care booklets as compensation. The survey included the following sections.

### 2.3 Anxiety and Depression Status

Hospital Anxiety and Depression Scale (HADS) was employed to evaluate patients’ anxiety and depression status during hospitalization in this study. After signing informed consent, research nurse conducted this survey at admission. HADS was specifically created to identify anxiety and depression symptoms in patients with physical health problems within a hospital setting. HADS has widely used in clinical trials and research studies to measure psychological distress as an outcome variable. It can effectively assess psychological distress without being influenced by somatic symptoms that might be due to the physical illness itself [16].

The HADS consists of 14 items and is divided into two subscales: (1) Anxiety Subscale (HADS-A): Seven Assessments to Measure Anxiety. (2) Depression Scale (HADS-D): Seven items that assess depression. Each item has an evaluation range from 0 to 3, where 0 indicates the lack of symptoms and 3 indicates the highest severity of symptoms. Scores for each subscale are evaluated as below 7 indicating no clinical anxiety or depression, and above 7 indicating the presence of anxiety or depressive symptoms [16]. The HADS does not assign a diagnostic diagnosis but rather assesses the severity of symptoms.

### 2.4 Sociodemographic and Clinical Variables

Sociodemographic characteristics, including age, sex, educational achievement, marital status, and health insurance status, were gathered using a standardized questionnaire. Clinical factors including the administration of HBV antiviral medication, albumin levels, prealbumin levels, total bilirubin, alanine aminotransferase and aspartate aminotransferase were recorded upon admission. This study identified sociodemographic and clinical variables based on their relevance in clarifying mental health outcomes in individuals with CHB. Sociodemographic factors were utilized due to their recognized influence on mental health outcomes. Gender is associated with increased anxiety levels, with women demonstrating greater vulnerability than men. Marital status and educational attainment were considered indicators of social support and coping ability, which can affect stress management. Liver function tests were chosen to assess the severity of CHB. We suggest that compromised liver function may exacerbate psychological distress, since patients face both physical challenges and uncertainty over their prognosis.

### 2.5 Data Analysis

Statistical analyses were performed utilizing Stata version 17.0 (StataCorp LLC, College Station, TX, USA). Descriptive statistical methods (e.g., frequencies, percentages, means, and standard deviations) were employed to evaluate the sociodemographic characteristics, as well as the anxiety and depression state of patients. The Chi-square test was used to compare groups based on categorical factors, including sex, marital status, education level, health insurance status, initial hospitalization, antiviral therapy, and liver-related illnesses. Non-normally distributed variables, including length of hospitalization, serum albumin, prealbumin, bilirubin, alanine aminotransferase (ALT), and aspartate transferase (AST), were presented as median (Interquartile Range, IQR) and analyzed using the Mann–Whitney U test, whereas continuous variables with a normal distribution, including age, anxiety, and depression scores, were expressed as Mean (M) ± Standard Deviation (SD) and compared using independent samples* t*-tests. A logistic regression model was employed to find factors linked with the HADs score. All variables were incorporated into the model. A *p* < 0.05 was considered statistically significant.

## 3. Results

### 3.1 Participant Characteristics, Anxiety and Depression Status

The majority of participants were male (n = 477, 73.5%) and currently married (n = 575, 88.6%), with a mean age of 47.6 years (SD = 12.95). Thirty percent of the participants possessed a college degree or above. 60.6% of participants were first-time admissions, whereas 94.9% had health insurance coverage.

According to the HADS score, 34.1% of subjects (n = 221) showed anxiety, while 71.7% (n = 465) showed symptoms of depression. The mean scores were 6.447 (SD = 3.247) and 9.253 (SD = 3.829), respectively. Table 1 shows the characteristics of the subjects, as well as their anxiety and depression states.

**Table 1.** **The participants’ characteristics, anxiety and depression status (N = 649)**.

Characteristics		N (%), M ± SD						
		All	Anxiety		*p*	Depression		*p*
		N = 649	>7	≤7		>7	≤7	
			221 (34.05)	428 (65.95)		465 (71.65)	184 (28.35)	
Sex					0.05			0.52
	Male	477 (73.50)	152 (31.80)	325 (68.13)		345 (72.33)	132 (27.67)	
	Female	172 (26.50)	69 (40.12)	103 (59.88)		120 (69.77)	52 (30.23)	
Age		47.59 ± 12.95	47.41 ± 13.67	47.68 ± 12.57	0.81	48.71 ± 13.02	44.76 ± 12.33	<0.001
Marital status					0.96			0.58
	Single	74 (11.40)	25 (33.78)	49 (66.22)		51 (68.92)	23 (31.08)	
	Married	575 (88.60)	196 (34.05)	379 (65.91)		414 (72.00)	161 (28.00)	
Education					0.41			0.03
	Under College	454 (70.00)	150 (33.04)	304 (66.96)		337 (74.23)	117 (25.77)	
	College	195 (30.0)	71 (36.41)	124 (63.59)		128 (65.64)	67 (34.36)	
Health Insurance					0.78			0.03
	Yes	616 (94.92)	209 (33.93)	407 (66.07)		436 (70.78)	180 (29.22)	
	No	33 (5.08)	12 (36.36)	21 (63.64)		29 (87.88)	4 (12.12)	
First Hospitalization					0.63			<0.001
	Yes	393 (60.60)	131 (33.33)	262 (66.67)		253 (64.38)	140 (35.62)	
	No	256 (39.40)	90 (35.16)	166 (64.84)		212 (82.81)	44 (17.19)	
Anxiety Score		6.447 ± 3.247	9.90 ± 1.93	4.67 ± 2.18	<0.001	7.16 ± 2.89	4.65 ± 3.41	<0.001
Depression Score		9.253 ± 3.829	9.96 ± 3.02	8.89 ± 4.14	<0.001	11.08 ± 2.63	4.63 ± 2.09	<0.001

### 3.2 Clinical Parameters Related to HBV Infection

**Fig. 1.**
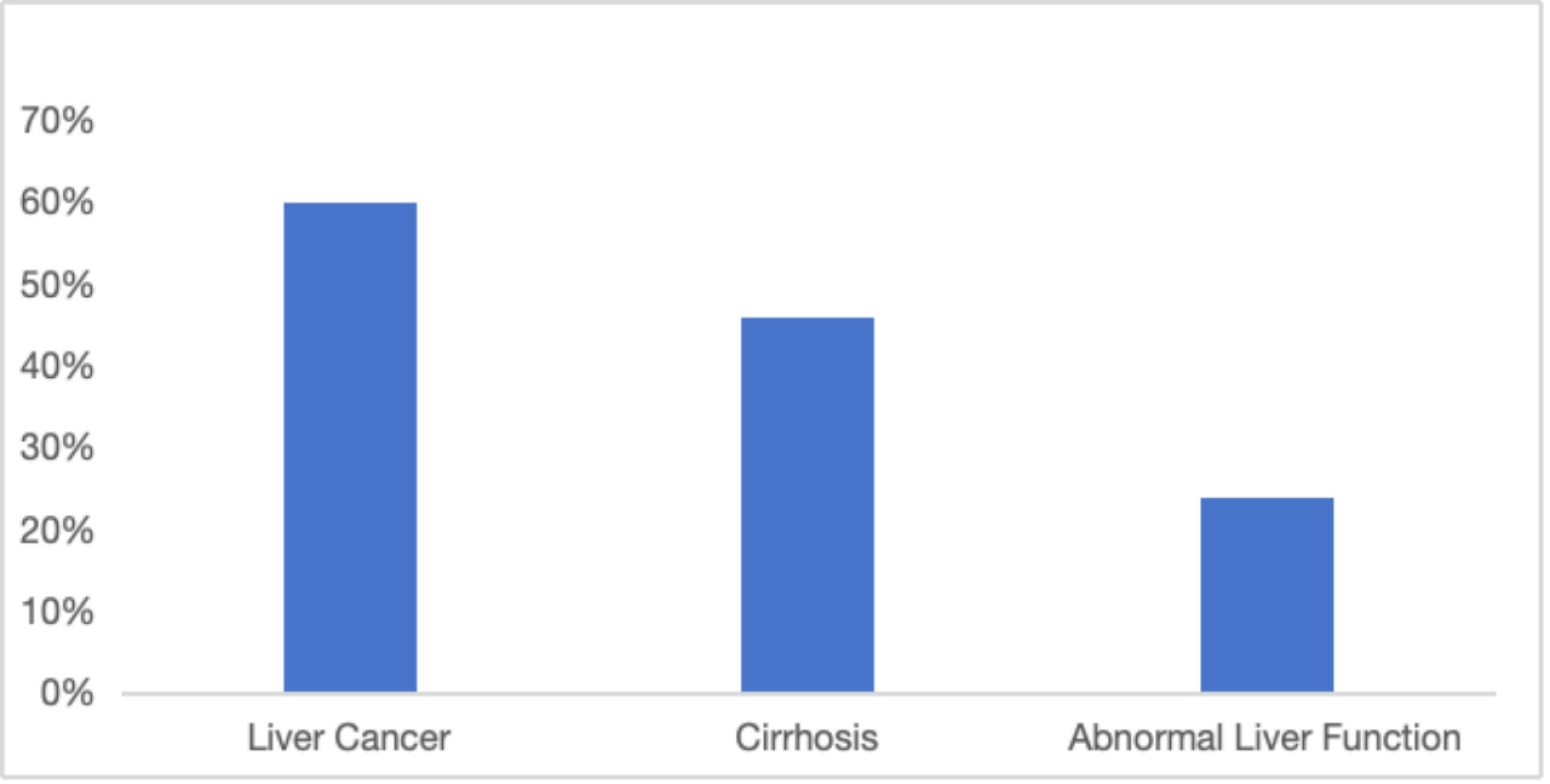
**Re-admission rate in patients with different diagnoses**.

Approximately 45% of the subjects were undergoing antiviral therapy upon admission. The majority of patients (n = 279, 43.0%) were diagnosed with abnormal liver function attributable to hepatitis B infection, followed by cirrhosis (n = 237, 36.5%) and liver-related cancer (n = 133, 20.5%). The median length of hospitalization was 7 days (IQR = 4–11). Additional clinical indicators, such as albumin, prealbumin, total bilirubin, alanine aminotransferase, and aspartate aminotransferase, are presented in Table 2. As the severity of the disease escalates, the incidence of patient re-admissions to the hospital correspondingly increases; the re-admission rates for patients with liver cancer, cirrhosis, and abnormal liver function diagnoses were 60%, 46.0%, and 24.0%, respectively (Fig. 1).

**Table 2.** **The relationship between clinical parameters and psychological distress (N = 649)**.

Parameters		N (%), M ± SD/Median (IQR)						
		All	Anxiety		*p*	Depression		*p*
		N = 649	>7	≤7	<0.001	>7	≤7	<0.001
			221 (34.05)	428 (65.95)		465 (71.65)	184 (28.35)	
Antiviral Therapy					0.447			0.059
	Yes	292 (45.00)	104 (35.62)	188 (64.38)		220 (75.34)	72 (24.66)	
	No	357 (55.00)	117 (32.77)	240 (67.23)		245 (68.63)	112 (31.37)	
Diagnosis					0.48			0.67
	Liver Cancer	133 (20.50)	43 (32.33)	90 (67.67)		99 (74.44)	34 (25.56)	
	Cirrhosis	237 (36.50)	79 (33.33)	158 (66.67)		166 (70.04)	71 (29.96)	
	Abnormal Liver Function	279 (43.00)	99 (35.48)	180 (64.52)		200 (71.68)	79 (28.32)	
Length of Hospitalization (Day)		7.0 (4.0, 11.0)	8.0 (4.0, 13.0)	6.0 (4.0, 10.0)	<0.001	8.0 (5.0, 12.0)	5.0 (3.0, 8.0)	<0.001
Serum Albumin		37.0 (32.0, 44.0)	36.0 (31.0, 43.0)	37.0 (33.0, 44.0)	0.15	38.0 (33.0, 45.0)	36.0 (31.0, 41.0)	0.15
Serum Prealbumin		140.0 (100.0, 180.0)	135.0 (95.0, 170.0)	142.0 (105.0, 185.0)	0.26	145.0 (100.0, 190.0)	138.0 (95.0, 175.0)	0.26
Serum Total Bilirubin		40.0 (20.0, 60.0)	42.0 (22.0, 62.0)	38.0 (18.0, 58.0)	0.72	42.0 (20.0, 61.0)	37.0 (17.0, 55.0)	0.72
Alanine aminotransferase (ALT)		80.0 (40.0, 160.0)	85.0 (45.0, 180.0)	75.0 (38.0, 150.0)	<0.001	90.0 (45.0, 185.0)	70.0 (35.0, 140.0)	<0.001
Aspartate transferase (AST)		70.0 (35.0, 145.0)	80.0 (40.0, 160.0)	65.0 (30.0, 130.0)	0.07	75.0 (38.0, 150.0)	60.0 (30.0, 120.0)	0.07

IQR, Interquartile Range.

### 3.3 Regression Analysis

Table 3 displays the results of the multivariate logistic regression analysis for the states of anxiety and depression in CHB patients. Gender influenced anxiety in this sample, with females demonstrating a greater propensity for anxiety. Nonetheless, depression has supplementary contributory factors. Advanced age, lack of health insurance, readmission, and extended hospitalizations were associated with depression.

**Table 3.** **Multiple logistic regression model of anxiety and depression in CHB inpatient (N = 649)**.

	Anxiety			Depression		
	Odds Ratio	*p*	(95% CI)	Odds Ratio	*p*	(95% CI)
Female	1.540	0.023	1.061–2.235	1.068	0.756	0.703–1.623
Age	0.999	0.930	0.985–1.014	1.019	0.026	1.002–1.036
Without Health Insurance Payment	1.137	0.732	0.545–2.374	3.415	0.031	1.119 –10.423
Un-Married	1.115	0.707	0.634–1.961	0.711	0.277	0.384–1.316
Do not on Antiviral therapy	0.816	0.246	0.578–1.151	0.790	0.229	0.538–1.160
College	1.160	0.454	0.787–1.710	0.873	0.528	0.574–1.329
Re-Admissions	1.134	0.710	0.803–1.602	2.526	<0.001	1.670–3.820
Length of Stay (Day)	1.021	0.069	0.998–1.043	1.064	<0.001	1.027–1.102
Serum albumin	1.002	0.761	0.989–1.015	0.995	0.478	0.982– 1.009
Serum prealbumin	1.001	0.283	0.999–1.003	1.002	0.198	0.999–1.004
ALT	1.000	0.517	0.999–1.000	0.999	0.190	0.999–1.000
AST	1.001	0.135	1.000–1.002	1.000	0.991	0.999–1.001
TB	1.000	0.670	0.998–1.002	1.000	0.685	0.997–1.002

TB, total bilirubin; CHB, chronic hepatitis B.

## 4. Discussion

### 4.1 Prevalence of Anxiety and Depression in CHB Inpatients

Using the HADS assessment tool, our study assessed anxiety and depression in hospitalised patients with chronic hepatitis B. To the best of our knowledge, this study contributes to the existing literature exploring the associated factors of anxiety and depression in patients with CHB, especially those in hospital. These findings can help healthcare professionals identify hospitalized patients at higher risk for anxiety and depressive symptoms, thereby providing empirical evidence to inform strategies to address mental health disorders in this population.

The study showed that 34.1% of hospitalized patients with CHB in Shenzhen suffered from anxiety, but disturbingly, 71.7% of patients showed symptoms of depression. The prevalence in our study significantly exceeded that in most outpatient settings, anxiety and depression were prevalent but not to this extent; the incidence of depression increased significantly in our study. Sirinimnualkul *et al*. [17] observed a prevalence of 10.2% for anxiety and 11.1% for depression among outpatients at a liver clinic. Mamo [18] indicated a prevalence of 23.6% for anxiety and 24% for depression among CHB patients, emphasizing that psychological illnesses are significantly more prevalent in inpatient environments than in outpatient settings. This aligns with the findings of studies which suggested that hospitalization worsening situation of anxiety and depression in chronic disease patients [19, 20], highlighting the significance of mental health issues in this population.

Furthermore, Wen-Tao *et al*. [21] also indicated that anxiety and depression often occur together, and was related to patient’s history of frequent hospitalization. Several variables likely contribute to the heightened frequency in an inpatient environment in Shenzhen. The severity of sickness and the acute need for medical care could heighten feelings of anxiety and depression, particularly in a hospital environment where patients face protracted stays and the stress of unfamiliar surroundings. Furthermore, the socio-economic factors peculiar to Shenzhen, a highly developed metropolis, may aggravate the mental health burden on these patients. Urban living is often linked to heightened stress due to rising living costs, demanding work conditions, and the necessity to retain employment while unwell. For many patients, hospitalization necessitates a leave of absence, often forcing them to disclose their chronic hepatitis B status to employers, which may lead to stigma and discrimination because of the disease’s contagious nature.

The significantly higher prevalence of depression compared to anxiety is particularly noteworthy. This disparity may be ascribed to various sources. Depression is often linked to chronic illness due to the ongoing demands of managing a long-term health condition, the perceived loss of control over one’s health, and the potential social isolation caused by the illness [22, 23, 24]. The stigma associated with CHB may intensify depressive symptoms, especially in Shenzhen, where social prestige and professional success are highly valued. In contrast, anxiety, while prevalent, is typically more transient and contingent upon specific circumstances, triggered by acute stressors such as hospitalization or medical procedures, as opposed to the persistent, pervasive impacts of CHB [25, 26, 27].

### 4.2 Associated Factors Identified in Regression Analysis

Regression analysis identified several factors that were significantly associated with depression, including age, repeated hospitalizations, and lack of health insurance, while gender was associated with anxiety. Women are more likely to experience anxiety, which is consistent with most of the literature on gender differences in mental health [28, 29, 30]. Recent research indicates that women have heightened sensitivity to low concentrations of corticotropin-releasing factor, a hormone that orchestrates stress reactions in mammals, rendering them twice as susceptible as males to stress-related illnesses [31]. Metacognitive views on uncontrollability, the benefits of worry, and avoidance may account for the increased prevalence of anxiety in females compared to males [32]. Furthermore, female patients with communicable diseases may encounter heightened social pressures and stigma, as they frequently assume the responsibility of managing both their health and the health and well-being of their families, thereby exacerbating their stress and anxiety in coping with a chronic illness such as CHB.

The notable prevalence of depression among older patients and those with recurring admissions contradicts findings suggesting that younger individuals are more vulnerable [33, 34]. These findings demonstrate that factors such as social media pressure, financial strain, and educational challenges significantly increase the vulnerability of younger individuals to depressive disorders. The high prevalence of depression in older CHB inpatients can be attributed to the chronic nature of the disorder, as older adults often experience accumulated psychological stress and heightened health-related worries over time. Our study demonstrates that as illness severity intensifies, the rate of patient re-admissions to the hospital correspondingly rises, showing a correlation between disease development and the frequency of hospital admissions. It is reasonable to deduce that patients readmitted to the hospital demonstrate more severe symptoms, either due to illness advancement or complications requiring more intervention. The psychological impact of repeated hospitalizations can be substantial, often intensifying feelings of hopelessness and anxiety about the future. Patients with recurrent hospitalizations may develop a concept of chronicity concerning their disease, perceiving their health as consistently deteriorating, which can significantly intensify depression symptoms [35]. This contrasts with first hospitalizations, during which patients may maintain hope for recovery or improvement, potentially alleviating profound depression.

The absence of health insurance was significantly correlated with depression, indicating that financial stress may exacerbate the mental health issues encountered by these patients, especially in a high-cost urban setting such as Shenzhen. This conclusion corresponds with research highlighting the significance of financial security in mental health [36, 37], while also indicating the necessity for region-specific healthcare policies that tackle the socio-economic issues encountered by CHB patients.

### 4.3 No Effects on Anxiety or Depression: Albumin, Prealbumin, and Other Clinical Parameters

This study examined the relationship between mental health and clinical parameters, including albumin, prealbumin, and liver function markers such as ALT, AST, and TB. Although previous studies [38, 39, 40, 41] have demonstrated that reduced levels of albumin and prealbumin—indicators of poor nutritional status and impaired liver function—as well as abnormal liver function markers are associated with negative mental health outcomes, our research found no significant correlation between these clinical parameters and the prevalence of anxiety or depression in CHB inpatients.

Our study reveals no significant correlation that can be ascribed to other variables. The mental health burden of our patient population may be significantly influenced by psychosocial factors, including hospitalization stress, stigma-related anxiety, and socioeconomic pressures. In advanced metropolitan settings like Shenzhen, environmental pressures may surpass the influence of clinical factors on mental health.

The diversity in the severity of liver disease among patients may potentially be a contributing factor. Nearly fifty percent of the patient cohort mostly comprised persons with stable or compensated liver disease in our study; hence, the impact of albumin and prealbumin on mental health may be less significant. Conversely, in individuals with advanced liver illness, where malnutrition and hepatic dysfunction are pronounced, these factors may exert a more substantial influence on mental health.

Moreover, the measures administered during hospitalization, including nutritional support and medical care, may have alleviated the adverse effects of inadequate nutritional status on mental health outcomes. Inpatients in a developed city such as Shenzhen may benefit from superior medical resources, perhaps diminishing the impact of these clinical factors on their mental health.

Ultimately, an alternate explanation for this discrepancy may be the focus on health education in China. In many cases, education regarding disease parameters is not prioritized over lifestyle and dietary education, hence limiting the patient’s understanding of the relationship between clinical markers and their mental health. This omission may lead to a reduced correlation between patient understanding of clinical parameters and mental health. Furthermore, this may suggest that other factors, such as psychosocial stressors and hospitalization settings, may have profound effects on the mental health outcomes of these patients.

### 4.4 Psychosocial and Psychological Factors Mediating Mental Health Outcomes

Psychosocial factors contribute to the elevated incidence of anxiety and depression among CHB inpatients, with psychological mechanisms mediating these effects. Chronic conditions such as hepatitis B can activate cognitive and emotional mechanisms that profoundly affect mental health.

Cognitive processes, including health-related anxiety and sickness perceptions, substantially affect anxiety and depression in this demographic. Research indicates that adverse perceptions of the condition, such as perceiving it as unmanageable or life-threatening, correlate with elevated levels of anxiety and depression [42, 43]. A sense of control or optimism may serve as a protective factor against certain mental health concerns.

Emotional processes like fear, embarrassment, and frustration can intensify the psychological burden of CHB. The stigma surrounding hepatitis B sometimes engenders guilt, leading to social isolation and depressive symptoms [44]. In urban areas like Shenzhen, where social stratification is pronounced, this distress is intensified by financial pressures arising from medical costs, especially for the uninsured.

Additionally, repeated hospitalizations may foster feelings of hopelessness and helplessness, which are central to depression. Patients may become ensnared in a loop of disease progression, intensifying emotional distress. The notion of the learned helplessness theory [45] suggests that prolonged exposure to uncontrollable stimuli can engender a sense of powerlessness in individuals, hence contributing to the development of depression.

### 4.5 Clinical Implications and the Impact of Urban Living

Due to the high frequency of anxiety and grief, it is important for healthcare providers to incorporate mental health assessments into routine care procedures for CHB hospitalized patients. The study highlighted key characteristics, such as gender, which revealed that female patients were more sensitive to anxiety and age, as older patients were at higher risk of depression. The lack of health insurance and frequent hospitalizations were substantially associated with depression, suggesting that financial instability and disease progression exacerbate mental health issues. Interventions must be customized to target specific risk factors while considering the socio-economic environment of patients.

Furthermore, addressing cognitive errors and emotional elements such as stigma and shame is essential in therapeutic interventions to enhance mental health results. Clinical management should thus integrate measures to mitigate these cognitive distortions, such as cognitive-behavioral therapy (CBT), to recontextualize negative beliefs of illness and alleviate the emotional burden. Interventions aimed at diminishing stigma and enhancing patients’ emotional coping strategies are expected to improve mental health outcomes and overall well-being for CHB inpatients.

The findings indicate that urbanization and rapid urban expansion may pose distinct stressors that must be addressed in relation to chronic diseases such as CHB. This study illustrates that socio-economic pressures, especially for uninsured individuals, can result in financial distress, exacerbating depression. Healthcare policy and patient support systems in metropolitan environments must confront these limitations, providing tailored assistance to alleviate the effects of financial instability on mental health.

## 5. Conclusion

This study elucidates the intricate relationship between physical and mental health in CHB patients within a highly developed urban environment. The findings necessitate a holistic approach to patient care that encompasses both the medical and psychological requirements of this demographic, ensuring that patients have the essential assistance to navigate the concurrent challenges of chronic hepatitis B and mental health disorders.

Understanding that clinical markers like albumin and prealbumin may not accurately reflect mental health status in this population can aid healthcare practitioners in prioritizing psychological and social interventions.

Incorporating mental health care into the overall treatment plan for CHB patients, especially in high-stress urban settings, allows healthcare professionals to improve both mental and physical health outcomes for these individuals. This holistic strategy may improve drug adherence and boost quality of life, as patients receive support for the full spectrum of challenges associated with managing their chronic condition.

## 6. Limitations

This study provides substantial insights into the mental health burden of CHB inpatients in Shenzhen, while several limitations should be acknowledged.

The cross-sectional method limits our ability to establish causal relationships between identified variables and mental health outcomes. Longitudinal studies are crucial for assessing the evolution of these connections over time and determining whether the diagnosed mental health problems are temporary or permanent.

Second, the study was conducted in remote urban areas, which limits the generalizability of the findings to other regions, especially rural areas where socioeconomic conditions, access to health care, and other factors may vary widely. The unique socioeconomic conditions in Shenzhen may have resulted in a higher prevalence of anxiety and depression, which may not fully represent the circumstances in other impoverished or rural areas of China.

A control group of non-HBV hospitalized patients was not included in this survey, which will provide insight into specific mental health issues associated with HBV infection compared to other chronic conditions. Future research should employ such controls to more accurately delineate the mental health impacts of HBV.

Notwithstanding these constraints, the study provides significant early data regarding the mental health state of CHB inpatients, highlighting the necessity for holistic care strategies that encompass both the physical and psychological dimensions of the disorder.

## Availability of Data and Materials

The datasets used or analyzed during this study are available from the corresponding author on reasonable request.

## Declaration of AI and AI-Assisted Technologies in the Writing Process

We would like to provide a clarification regarding the high AI detection rate associated with our manuscript. Our authorship did not utilize any AI writing tools during the preparation and writing of the manuscript. However, as our native language is Chinese, we used the Youdao Translation software (a European-Chinese translation tool) to assist in translating the content into English for submission. After using this tool, the authors reviewed and edited the content as needed and takes full responsibility for the content of the publication.
